# Influence of DOTA Chelators on Radiochemical Purity and Biodistribution of ^177^Lu- and ^90^Y-Rituximab in Xenografted Mice

**Published:** 2018

**Authors:** Urszula Karczmarczyk, Wioletta Wojdowska, Renata Mikołajczak, Michał Maurin, Ewa Laszuk, Piotr Garnuszek

**Affiliations:** *National Centre for Nuclear Research, Radioisotope Centre POLATOM, Otwock, Poland.*

**Keywords:** Anti-CD20, ^177^Lu and ^90^Y, Radiolabeling, Rituximab, DOTA chelator, Animal study

## Abstract

This work presents a comparative biological evaluation of ^90^Y- and ^177^Lu- labelled DOTA-SCN and DOTA-NHS conjugated to Rituximab in tumour-bearing mice. Two DOTA derivatives, p-SCN-Bn-DOTA and DOTA-NHS-ester were conjugated to Rituximab and then freeze-dried kit formulations were prepared, as previously described (1). Tissue distribution was investigated in tumour-bearing (Raji s.c.) male Rj: NMRI-Foxn1^nu^/Foxn1^nu^ mice at different time points after administration of ^177^Lu-DOTA-Rituximab or ^90^Y-DOTA-Rituximab (6 MBq/10 μg per mouse). In addition, tumour images were acquired with a PhotonIMAGER^TM^ after injection of ^90^Y-DOTA (SCN)-Rituximab. All radioimmunoconjugates were obtained with high radiolabelling yield (RCP > 98%) and specific activity of ca. 0.6 GBq/mg. The conjugates were stable in human serum and in 0.9% NaCl; however, progressive aggregation was observed with time, in particular for DOTA -(SCN) conjugates. Both ^177^Lu- and ^90^Y-DOTA -(SCN)-Rituximab revealed slow blood clearance. The maximum tumour uptake was found 72 h after injection of ^177^Lu-DOTA -(SCN)-Rituximab (9.3 ID/g). A high radioactivity uptake was observed in liver and spleen, confirming the hepatobiliary excretion route. The results obtained by the radioactive optical imaging harmonize with those from the biodistribution study.

## Introduction

Rituximab, a chimeric mouse/human monoclonal antibody (mAb) (2, 3, 4) coupled with beta emitting radionuclides, enhances therapeutic effectiveness of anti-CD20 (5, 6, 7). Such radioimmunoconjugates bind to CD20 antigen, a transmembrane phosphorylated protein, located on pre-B and matured B-lymphocytes and more than 90% of B-cell Non-Hodgkin’s Lymphomas (NHL) (8, 9, 10).

Pre-clinical and clinical applications of ^131^I- (11), ^99m^Tc- (12), ^90^Y- (13, 14, 15), or ^177^Lu-labeled (16) Rituximab have been already reported. Many reports described the radiolabelling of anti-CD20 with ^177^Lu and ^90^Y after conjugation with DOTA or DTPA as a bifuncional chelating agents because they form very stable complexes (17, 18). However, chemical modification of antibodies may alter their chemical or biological properties. Based on the recent experiments on the kit formulation of DOTA-Rituximab conjugate (1), our present study is focused on comparative biodistribution of radioimmunoconjugates prepared after conjugation of Rituximab with DOTA-(SCN) or DOTA (NHS) chelator. The biodistribution is an essential preclinical procedure to assess the localization of radioligand. It allows determining the off-target accumulation and clearance rate (19). To achieve this, organs and tissues are excised from animals (usually mice and rats) at indicated time points and analyzed. This is laborious and involves the use of many animals. In order to preliminarily and non-invasively assess the ^90^Y-DOTA-Rituximab biodistribution *in-vivo*, we used optical imaging. Numerous studies have demonstrated that for a variety of β^+^, β^-^ radionuclides bound up with peptides, antibodies and nanoparticles enable optical imaging due to Cerenkov light emission (20, 21).

Herein we report the influence of two DOTA precursors, p-SCN-Bn-DOTA and DOTA-NHS-ester conjugated to Rituximab and radiolabelled with ^177^Lu and ^90^Y on radiochemistry and biodistribution in tumour-bearing mice.

## Experimental

*Antibody and reagents*

Rituximab (MabThera®) was purchased from Roche Pharma AG, Germany. The bifunctional chelators: p-SCN-Bn-DOTA(2-(4-isothiocyanatobenzyl)-1,4,7,10-tetraazacyclododecane-1,4,7,10-tetraacetic acid) and DOTA-NHS-ester (1,4,7,10-Tetraazacyclododecane-1,4,7,10-tetraacetic acid mono (*N*-hydroxysuccinimide ester) were purchased from Macrocyclics. Both radionuclides in the chemical form of chloride in 0.04 M HCl, i.e.: Lutetium-177 (LutaPol) of SA higher than 555 MBq/mg Lu, and non-carrier added Yttrium-90 (ItraPol) were produced at Radioisotope Centre POLATOM, Poland. All other chemicals and materials were used as supplied and were of the highest purity available. For all procedures HPLC grade water was used to avoid metal contamination.

*Synthesis of the DOTA-Rituximab conjugates*

Two derivatives of DOTA bifunctional chelating agent, i.e. DOTA-SCN and DOTA-NHS, were conjugated to anti-CD20 antibody (Rituximab) and finally the conjugates were formulated in the form of a freeze-dried kit as previously described (1). Briefly, the concentrated Rituximab (20 mg in 2 mL) was incubated with a 10-fold molar excess of DOTA-SCN chelator at 37 °C for 1.5 h in the carbonate buffer with gentle mixing. An excess of the unreacted chelator was removed by ultrafiltration (Amicon Ultra; MWCO 30,000; Millipore). For preparation of Rituximab conjugate with DOTA-NHS-ester the protocol developed within the IAEA project with slight modifications was used (22). The solution of anti-CD20 (Rituximab; 10 mg/mL) was initially purified by ultrafiltration using an Amicon centrifuge filter Ultra-2mL (30 min, 5000 rpm). The concentrated mAb was incubated with 40mM of DTPA at 4 °C for 30 min and then loaded on the Sephadex G-25 column (PD-10) and eluted with a 0.05 M phosphate buffer of pH 7.0. The 0.5 mL fractions with the highest concentration of mAb (quantified calorimetrically with the Bradford method) were collected and incubated with 100-fold molar excess of DOTA-NHS-ester at 4 °C with gentle stirring for 24 h. The reaction mixtures were transferred on an Amicon centrifuge filter to exchange the buffer to 0.5 M ammonium acetate of pH 5.5 and to remove the excess of unbound DOTA-chelator. The concentration of the antibody in the final immunoconjugate solution was measured by the Bradford method. The average number of DOTA molecules coupled per mAb molecule for both chelators were determined by radiolabelling assay using ^64^Cu (23, 24).

*Radiolabelling*

The immunoconjugates were radiolabeled with ^177^Lu and ^90^Y as follows: the freeze-dried kit containing 2.0 mg of DOTA-anti-CD20 was dissolved in 0.5 mL of water and mixed with 100-900 MBq of [^177^Lu]LuCl_3_ or ^90^YCl_3_. The solutions were incubated at 37 °C for 1h. The quality control of the resulting radioimmunoconjugates was performed by ITLC-SG with methanol/0.4 M ammonium acetate (1:1, v/v) as a mobile phase and by size exclusion high performance chromatography (SE-HPLC) using BioSep-SEC-S3000 PEEK column (300x7.5 mm; Phenomenex) eluted with a 0.1 M phosphate buffer of pH 5.8 at a flow rate of 1 mL/min. The radiochemical purity (RCP) and stability of ^177^Lu-DOTA-Rituximab and ^90^Y-DOTA-Rituximab were evaluated in the presence of an excess of competitor such as 10mM DTPA. Due to a high RCP of the ^90^Y- and ^177^Lu- DOTA-Rituximab obtained, no additional purification step was necessary.

Stability of ^90^Y-DOTA-Rituximab and ^177^Lu-DOTA-Rituximab in human serum and in 0.9 % NaCl was determined after 1, 24, 48, and 72 h of incubation using SE-HPLC and ITLC-SG radioanalytical techniques. To investigate stability in human serum, 200 µL of ^177^Lu or ^90^Y-immunoconjugates were incubated in 0.5 mL of fresh human serum up to 72 h at 37 °C.

*Cell culture*

The Raji cells were purchased from American Type Culture Collection (ATCC no. CCL-86). The cells were cultured in RPMI 1640 medium supplemented with 10% heat-inactivated fetal bovine serum (Gibco) and antibiotics (penicillin 100 U/mL, and streptomycin 100 μg/mL). The cells were cultured in 5% CO_2_ at 37 °C and passaged every 2 days. The cells in logarithmic growth phase were used for *in-vivo* studies. 

*Biodistribution studies*

All animal experiments were approved by the 4th Local Animal Ethics Committee in Warsaw (authorization number 34/2013), and were carried out in accordance with the national legislation regarding laboratory animals protection and the principles of good laboratory practice.

The human lymphoma cell lines (Raji) was used for xenografts. Male Rj: NMRI-Foxn1^nu^/Foxn1^nu^ mice (from Janvier Lab. France) were subcutaneously grafted with 10^6^ cells (cell suspension in 200 μL of Matrixgel™ Basement Membrane Matrix (BD Sciences) on the left or right shoulder, under anesthesia with 2% isoflurane. The experiments were performed 2–3 weeks after implantation, when a tumour reached a volume of approximate 100–300 mm^3^. In a typical experiment, a groups of 3–4 mice were injected (tail vein) with 10 μg (6 MBq) of ^90^Y- and ^177^Lu- DOTA-Rituximab in 100 µL each. The animals were euthanized by overdose of isoflurane at 4, 24, 48, and 72 h post-injection (p.i.). Samples of blood and selected organs were collected, weighed and their radioactivity was measured by the NaI gamma counter supplied with adapter for the whole body measurement in case of luthetium-177. The organ uptake values were expressed as the percent of injected dose per organ (%ID) and per gram of tissue (%ID/g). Additionally, the mice injected with ^90^Y-DOTA-(SCN)-Rituximab were optically imaged at different time points using the PhotonIMAGER^TM^ System (Biospace LAB), based on the detection of Cerenkov emission.

*Data Analysis*

A two-way analysis of variance (ANOVA) performed with GraphPad Prism 5 for Windows, was applied for comparison of radioactive DOTA-Rituximab concentration in the blood, urine and selected organs in the different treatment groups from the biodistribution study. The Post-hoc Bonferroni analysis was applied for determination of the statistically significant relationships among the treatment groups. A *p*-value of < 0.05 determined statistical significance. All results were expressed as mean ± s.d.

## Results and Discussion

*Conjugation of chelating agents to anti-CD20 and radiolabelling*

The two DOTA derivatives were conjugated to Rituximab with an initial molar ratio 10:1 for SCN-DOTA and 100:1 for NHS-DOTA, which resulted in approximately 5 and 18 of the chelator molecules per the mAb, respectively. The DOTA-Rituximab conjugates were radiolabeled with ^177^Lu and ^90^Y with radiochemical yields ranging from 98% to 100% and being prepared with specific activity from 100 to 900 MBq/mg. The influence of specific activity on radiochemical purity is presented in tables 1 and 2 (where the first value is the percent of a sum of aggregates and monomer, while the second one, presented in brackets, corresponds to the percentage of monomer). For both radioimmunoconjugates, optimal specific activities as high as 600 MBq/mg have been reached under the same reaction conditions and the radiochemical yield ranging from 98.7% to 99.0% and from 98.8% to 99.1% at 1 h for ^177^Lu-DOTA-Rituximab and ^90^Y-DOTA-Rituximab respectively. The radioimmunoconjugates were stable in 0.9% NaCl and human blood serum for at least 48 h.

**Table 1:** Influence of specific activity on radiochemical purity of^ 177^Lu-DOTA (SCN)-Rituximab and ^177^Lu-DOTA (NHS)-Rituximab

**Specific activity [MBq/mg]**	**Radiochemical purity [%] at time (n = 2-3)**							
	^177^ **Lu-DOTA(SCN)-Rituximab**				^177^ **Lu-DOTA(NHS)-Rituximab**			
	1 h	24 h	48 h	72 h	1 h	24 h	48 h	72 h
100 - 300	99.7 ± 0.4 (94.7 ± 3.5)	98.7 ± 0.6 (90.2 ± 2.3)	96.4 ± 0.6 (86.8 ± 2.3)	95.4 ± 2.9 (81.1 ± 0.6)	98.2 ± 0.4 (93.4 ± 1.8)	95.9 ± 1.9 (92.7 ± 2.4)	93.8 ± 2.8 (88.4 ± 3.2)	94.9 ± 2.5 (89.0 ± 3.2)
301 - 600	99.0 ± 0.9 (94.3 ± 2.4)	96.6 ± 2.1 (83.2 ± 2.6)	90.5 ± 4.4 (80.0 ± 0.4)	85.4 ± 4.4 (78.6 ± 4.0)	99.2 ± 1.3 (96.7 ± 3.3)	95.6 ± 3.8 (92.2 ± 3.9)	95.5 ± 2.5 (89.2 ± 3.1)	94.4 ± 3.4 (89.3 ± 3.1)
601 - 900	99.0 ± 0.2 (92.4 ± 2.1)	97.2 ± 1.8 (79.0 ± 13.6)	95.1 ± 0.6 (74.2 ± 3.7)	86.1 ± 1.8 (59.9 ± 0.8)	98.7 ± 0.1 (95.4 ± 1.1)	95.0 ± 0.5 (91.6 ± 0.6)	92.5 ±1.1 (98.4 ± 0.8)	91.9 ± 0.8 (86.4 ± 1.1)

**Table 2:** Influence of specific activity on radiochemical purity of^ 90^Y-DOTA (SCN)-Rituximab and ^90^Y –DOTA (NHS)-Rituximab

**Specific activity [MBq/mg]**	**Radiochemical purity [%] at time (n = 2-3)**							
	^90^ **Y-DOTA(SCN)-Rituximab**				^90^ **Y-DOTA(NHS)-Rituximab**			
	1 h	24 h	48 h	72 h	1 h	24 h	48 h	72 h
100 - 300	98.9 ± 1.9 (98.3 ± 2.9)	96.0 ± 2.2 (85.8 ± 4.3)	92.5 ± 4.3 (76.3 ± 2.1)	84.1 ± 2.9 (77.8 ± 0.6)	98.3 ± 1.0 (93.2 ± 1.0)	97.3 ± 0.1 (93.0 ± 4.2)	93.3 ± 4.2 (88.0 ± 3.4)	89.7 ± 2.5 (81.9 ± 7.6)
301 - 600	96.6 ± 1.8 (95.3 ± 0.9)	89.8 ± 4.3 (76.6 ± 2.8)	90.3 ± 9.5 (72.7 ± 3.1)	75.4 ± 16.7 (78.6 ± 3.0)	96.7 ± 1.7 (91.3 ± 1.5)	93.3 ± 2.8 (87.5 ± 3.5)	90.0 ± 7.4 (83.3 ± 5.7)	85.2 ± 9.3 (76.5 ± 6.9)
601 - 900	99.1 ± 0.1 (96.6 ± 2.9)	89.5 ± 5.4 (76.9 ± 4.5)	84.5 ± 0.6 (69.5 ± 3.7)	82.4 ± 1.8 (73.1 ± 0.8)	98.8 ± 1.2 (94.0 ± 3.8)	94.2 ± 3.0 (91.8 ± 3.0)	89.3 ±0.1 (87.2 ± 1.2)	87.0 ± 2.8 (82.5 ± 4.7)

*Biodistribution in the tumour-bearing mice*

The results of the tissue distribution of ^177^Lu-DOTA-(SCN)–Rituximab and ^177^Lu-DOTA (NHS)–Rituximab conjugates are presented in table 3. The tumour uptake reached a value of 7.3 ± 1.7 %ID/g at 24 h for ^177^Lu-DOTA -(SCN)-Rituximab and increased to 9.3 ± 1.0 at 72 h p.i.v. In contrast, the tumour accumulation of ^177^Lu-DOTA-(NHS)-Rituximab reached the maximum value of 7.3 ± 1.7 %ID/g at 24 h and appears to be constant up to 72 h (6.9 ± 0.7 %ID/g). A relatively high accumulation of radioactivity was found in the blood at 4 h p.i.v. (20.1 ± 4.7 and 20.8 ± 1.0 for DOTA-(SCN) and DOTA-(NHS) respectively). However, the conjugates were quickly cleared from the blood stream and the radioactivity decreased to 7.0 ± 0.7 and 6.0 ± 0.9 at 48 h. 

The highest uptake was observed for blood-rich organs like spleen and liver. In the spleen 23.7 ± 1.7 %ID/g of ^177^Lu-DOTA-(SCN)-Rituximab at 4 h was found, which then increased to 27.9 ± 5.3 %ID/g at 48 h p.i.v. Similar results were obtained for ^177^Lu-DOTA-(NHS)-Rituximab: 18.7 ± 3.1 and 27.4 ± 1.8 %ID/g at 4 and 72 h, respectively.

**Table 3:** Biodistribution of the ^177^Lu-DOTA-Rituximab in tissue/organs collected at different time points in nude mice bearing s.c. Raji cell (n = 4-5, mean values %ID/g ± SD).

	^177^ **Lu-DOTA(SCN)-Rituximab**				^177^ **Lu-DOTA(NHS)-Rituximab**			
	4 h	24 h	48 h	72 h	4 h	24 h	48 h	72 h
Blood	20.1 ± 4.7	8.9 ± 3.2	7.0 ± 0.7	10.7 ± 1.9	20.8 ± 1.0	13.0 ± 1.7	6.0 ± 0.9	8.3 ± 1.8
Lung	9.2 ± 1.1	4.6 ± 1.1	2.8 ± 0.6	5.0 ± 0.3	9.8 ± 0.3	6.0 ± 0.2	3.4 ± 0.7	4.2 ± 0.8
Liver	17.0 ± 1.2	10.8 ± 2.0	8.3 ± 0.7	9.4 ± 1.3	15.8 ± 1.0	10.2 ± 1.7	7.4 ± 1.2	8.2 ± 0.8
Spleen	23.7 ± 1.7	19.2 ± 10.8	27.9 ± 5.3	17.6 ± 10.0	18.7 ± 3.1	28.7 ± 4.1	24.9 ± 2.9	27.4 ± 1.8
Kidneys	6.3 ± 1.9	4.4 ± 0.7	3.4 ± 0.5	4.5 ± 1.7	7.8 ±1.9	4.6 ± 1.1	3.7 ± 0.8	3.3 ± 0.5
Bone	8.6 ± 0.3	4.9 ± 0.6	5.9 ± 0.5	4.0 ± 1.0	4.9 ± 0.5	6.3 ± 2.3	5.6 ± 0.5	6.9 ± 2.0
Muscle	1.3 ± 0.6	0.9 ± 0.1	0.5 ± 0.1	1.0 ± 0.0	1.7 ± 0.3	1.2 ± 0.2	0.9 ± 0.1	0.9 ± 0.1
Tumour	1.9 ± 0.2	7.4 ± 1.4	5.8 ± 1.0	9.3 ± 1.0	2.7 ± 0.4	7.3 ± 1.7	6.9 ± 0.6	6.9 ± 0.7
Urine [%ID]	6.0 ± 1.1	14.8 ± 0.2	25.4 ± 1.7	27.8 ± 0.6	1.8 ± 0.4	8.8 ± 2.0	19.2 ± 1.5	28.3 ± 4.7

At the established time points, when ^177^Lu-DOTA-(SCN)-Rituximab was administered, the %ID urinary excretion was 6.0 ± 1.1, 14.8 ± 0.2, 25.4 ± 1.7 and 27.8 ± 0.6 while for ^177^Lu-DOTA (NHS)-Rituximab it was 1.8 ± 0.4, 8.8±2.0, 19.2 ± 1.5 and 28.3 ± 4.7 % ID at 4, 24, 48 and 72 h, respectively. The percentage of excreted radioactivity increased with time post injection for both chelators but the only statistically significant difference (*P *< 0.0001) was observed at 48 h.

Differences in bone uptake between DOTA-(SCN) and DOTA-(NHS)-Rituximab were observed at 4 h p.i. (8.6 ± 0.3 for ^177^Lu-DOTA-(SCN)-Rituximab and 4.9±0.5 for ^177^Lu-DOTA-(NHS)-Rituximab). However, in case of ^177^Lu-DOTA-(SCN)-Rituximab the radioactivity in bone significantly decreased to 4.0 ± 1.0 % ID/g at 72 h post injection and it was lower compared to the DOTA-(NHS) radioimmunoconjugates 6.9 ± 2.0 %ID/g.

**Figure 1:**
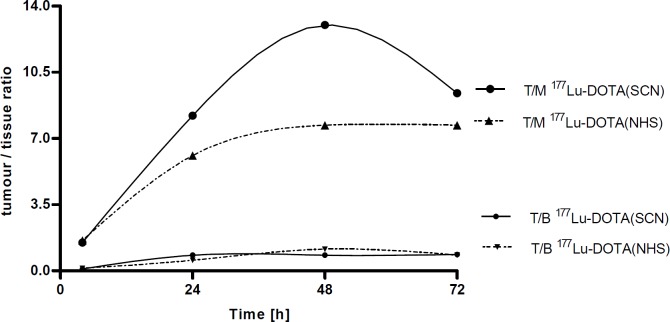
Changes of T/nT (tumour to non-tumour tissues: blood (T/B) and muscle (T/M)) ratio for ^177^Lu-DOTA (SCN)-Rituximab and ^177^Lu-DOTA (NHS)-Rituximab

The^177^Lu-DOTA-(SCN)-Rituximab conjugate demonstrated a higher tumour to muscle ratio (T/M), i.e. 1.5, 8.21, 13.0 and 9.4 at 4, 24, 48 and 72 h, respectively. However, it was not statistically significant (*P *> 0.05) compared to T/M observed for ^177^Lu-DOTA-(NHS)-Rituximab (1.6, 6.1, 7.7 and 7.7 at 4, 24, 48 and 72 h, respectively; Figure 1).

It was noted that for ^177^Lu-DOTA-(SCN)-Rituximab the T/nT tissue ratios reached maximum values of 0.8, 0.7, and 0.4 for blood, liver, and spleen, respectively at 24 h p.i. The tumour to bone ratio peaked to 2.3 at 72 h. The ^177^Lu-DOTA-(NHS)-Rituximab conjugate showed a little higher T/nT ratios for the blood-rich organs than for DOTA-(SCN) chelator but not statistically significant. 

*In-vivo* *Cerenkov imaging of *^90^*Y-DOTA-Rituximab*

**Figure 2:**
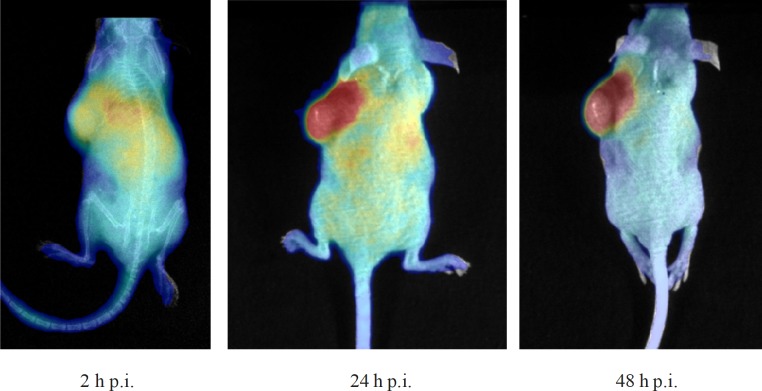
*In-vivo* Cerenkov optical imaging of mouse bearing a Raji xenograft following injection of ^90^Y-DOTA (SCN)-Rituximab (10 µg, 0.7 GBq/mg) at 2, 24 and 48 h after i.v. injection

The mice injected with ^90^Y-DOTA (SCN)-Rituximab were optically imaged at 2, 24 and 48 h p.i.v., (Figure 2). It could be clearly seen that radioimmunoconjugates were accumulated in the liver as well as in the spleen at 2 h p.i.v. The highest accumulation in tumour tissue was excellently visible in the optical images at 24 h p.i.v. The next 24 h observation showed decrease at non-target organs and retention of activity in the tumour. These observations in optical imaging corresponds with those from the biodistribution measurements.

## Conclusion

Previously we reported the development of a freeze-dried kit formulation for DOTA-(SCN)-Rituximab radiolabelling with lutetium-177 and yttrium-90 (1), which enabled convenient preparation of the radioactive solution for injection with yields higher than 98%. We also investigated bioconjugate’s stability in serum and 0.9% NaCl up to 48 h. The developed DOTA-(NHS)-Rituximab kit could be labelled straightforward with the radiochemical yield of more than 98% and additionally with less aggregation of the mAb then DOTA-(SCN)-Rituximab.

The biodistribution study in xenografted mice with ^177^Lu-DOTA-Rituximab showed that the main route of elimination was hepatobiliary tract and no statistically significant differences between the DOTA-(SCN) and DOTA-(NHS) conjugates were observed. The *in-vivo* stability of described radiolabelled complexes was confirmed by a low accumulation in bone, considering a high affinity of free ^177^Lu to bone tissue (25, 26). The increased uptake of ^177^Lu-DOTA-Rituximab over time was observed in tumour. We also observed a stable but low tumour/blood ratio c.a. 1 (Figure 1) which should be > 2 as described by Reilly (27). The bone/blood ratio increased with time (from 0.2 to 0.8 at 4 and 72 h p.i.v., respectively) for ^177^Lu-DOTA (NHS)-Rituximab but did not increase since 24 h p.i.v., for ^177^Lu-DOTA-(SCN)-Rituximab. It suggested a higher stability of the metal-ligand complexes of ^177^Lu-DOTA-(SCN)-Rituximab.

Optical imaging systems can be used for imaging in pre-clinical models. *In-vivo* Cerenkov imaging of ^90^Y-DOTA-Rituximab in tumour bearing mice confirmed accumulation of radioactivity in tumour.
